# Bacterial Community Succession, Transmigration, and Differential Gene Transcription in a Controlled Vertebrate Decomposition Model

**DOI:** 10.3389/fmicb.2019.00745

**Published:** 2019-04-18

**Authors:** Zachary M. Burcham, Jennifer L. Pechal, Carl J. Schmidt, Jeffrey L. Bose, Jason W. Rosch, M. Eric Benbow, Heather R. Jordan

**Affiliations:** ^1^Department of Biological Sciences, Mississippi State University, Starkville, MS, United States; ^2^Department of Entomology, Michigan State University, East Lansing, MI, United States; ^3^Department of Pathology, University of Michigan, Ann Arbor, MI, United States; ^4^Department of Microbiology, Molecular Genetics, and Immunology, University of Kansas Medical Center, Kansas City, KS, United States; ^5^Department of Infectious Disease, St. Jude Children’s Research Hospital, Memphis, TN, United States; ^6^Department of Osteopathic Medical Specialties, Michigan State University, East Lansing, MI, United States

**Keywords:** decomposition, postmortem microbiome, necrobiome, metatranscriptomic, metagenomic

## Abstract

Decomposing remains are a nutrient-rich ecosystem undergoing constant change due to cell breakdown and abiotic fluxes, such as pH level and oxygen availability. These environmental fluxes affect bacterial communities who respond in a predictive manner associated with the time since organismal death, or the postmortem interval (PMI). Profiles of microbial taxonomic turnover and transmigration are currently being studied in decomposition ecology, and in the field of forensic microbiology as indicators of the PMI. We monitored bacterial community structural and functional changes taking place during decomposition of the intestines, bone marrow, lungs, and heart in a highly controlled murine model. We found that organs presumed to be sterile during life are colonized by *Clostridium* during later decomposition as the fluids from internal organs begin to emulsify within the body cavity. During colonization of previously sterile sites, gene transcripts for multiple metabolism pathways were highly abundant, while transcripts associated with stress response and dormancy increased as decomposition progressed. We found our model strengthens known bacterial taxonomic succession data after host death. This study is one of the first to provide data of expressed bacterial community genes, alongside transmigration and structural changes of microbial species during laboratory controlled vertebrate decomposition. This is an important dataset for studying the effects of the environment on bacterial communities in an effort to determine which bacterial species and which bacterial functional pathways, such as amino acid metabolism, provide key changes during stages of decomposition that relate to the PMI. Finding unique PMI species or functions can be useful for determining time since death in forensic investigations.

## Introduction

Decomposing remains are a continuously shifting ecological system leading to changes in nutrient availability and microhabitat conditions, thus yielding a microbial consortium under constant selective pressures (Carter et al., 2007; Janaway et al., 2009; Hyde et al., 2013; Metcalf et al., 2016). Microorganisms associated with vertebrate remains are ubiquitous and may be deposited on a body from the environment, invertebrate, or vertebrate scavengers of the necrobiome or were part of the existing microflora during life (Benbow et al., 2018). Vertebrate decomposition begins immediately after death and as tissue begins to breakdown during autolysis, an efflux of cellular components and nutrients occurs which is used by microbial, predominately bacterial, communities (Janaway et al., 2009; Hyde et al., 2013; Crippen et al., 2015). After an initial lag phase immediately following organismal death, bacterial communities begin to exponentially proliferate, transmigrate, and create specialized proteins that digest host tissues during putrefaction (Can et al., 2014; Pozhitkov et al., 2017). These metabolic changes drive the transformation of the environmental decomposing landscape through the release of waste products, nutrient depletion, oxygen availability, and pH cycles which further facilitates host tissue breakdown. In turn, bacterial communities involved in the decomposition process are highly dynamic and constantly competing for survival, nutrient acquisition, and habitat space.

Bacterial community succession occurs in a predictive manner associated with the time since death, or postmortem interval (PMI) (Finley et al., 2015; Hauther et al., 2015; Burcham et al., 2016). This discovery has led to extensive research of bacterial communities associated with remains for understanding intrinsic microbial ecology of decomposition, and more recently, to aid in biomarker discovery for forensic PMI estimation (Pechal et al., 2014; Javan et al., 2016; Metcalf et al., 2016). PMI estimation supports forensic investigations by providing a window of time when death occurred to help support or refute eyewitness accounts regarding events leading up to death. These community analyses have now been widely performed on animal models targeting the bacterial 16S gene for taxonomic community profiling and predictive function in an attempt to discover PMI-associated biomarkers (Damann and Carter, 2013; Metcalf et al., 2013, 2016; Pechal et al., 2014). Although research is also conducted on human remains, animal models provide investigators the ability for robust sample sizes in order to create statistically powerful studies, and control of habitat conditions, such as with insect colonization, temperature, etc. (Finley et al., 2015; Hyde et al., 2017; Pechal et al., 2018). In a study using terrestrial swine models, microbial diversity decreased as decomposition progressed with Proteobacteria being the dominant phylum in early stages, while Firmicutes dominated late stages. In those studies, Clostridiaceae was one of the most dominant families toward the end of decomposition (Pechal et al., 2014). Additionally, in terrestrial murine models, during bloat stage the abdominal cavity has increased relative abundances of anaerobic gut microbiota, Lactobacillaceae, and Bacteroidaceae, but transitions to contain more oxygen-tolerant (i.e., Enterobacteriaceae) bacteria following burst (Metcalf et al., 2013, 2016). Overall, animal models have shown similarities to microbial succession discovered in human remains such as the transition from aerobic to anaerobic bacteria, prevalence of *Clostridium* in late stages, and bacterial community differences based on body location (Hyde et al., 2013; Finley et al., 2015; Hauther et al., 2015). These data are promising for the use of animal decomposition models as surrogates for microbial involvement within human remains, and for measuring correlates of microbial structure and function during decomposition.

While recognizing the broad trends in microbial contributions to decomposition is important for narrowing research foci, it is imperative that we study microbial interactions at a finer resolution with reference baseline activity, if specific, usable biomarkers are going to be detected. This fine resolution along with baseline data approach is important for teasing apart bacterial taxonomic and functional succession variability in the decomposition process, which may affect potential microbial biomarker discovery. This approach raises the call for highly controlled studies with the ability to focus on the microbial interactions solely associated with the host so that a host baseline can be established and built upon with other variables (i.e., climate, soil, and scavengers) and external environmental microbial communities.

Our study aims to develop the host postmortem microbial structure in conjunction with functional activity using a highly controlled laboratory setting, without the introduction of external environmental factors. We are also among the first to utilize postmortem microbial metatranscriptomic analyses. While metagenomic investigation provides insights about microbial community structure and functional potential, metatranscriptomic investigation is a useful tool to shed light on the active functional profiles of a microbial community. Together, both analytic methods provide knowledge on both the microbial diversity as well as genes actively involved in ecosystem processes. For instance, are postmortem microbiome changes observed and measured during decomposition succession reflective of coordinated responses, plasticity within an individual microbial species, or consequences of environment disturbance? Data have shown that many coexisting but taxonomically distinct microbes can encode genes for the same metabolic functions, which may blur the association between community composition and ecosystem functioning (Louca et al., 2018). However, for functions performed by only a few taxa, the sensitivity and resilience of this function may closely follow changes in the abundance of those taxa (Langenheder et al., 2006; Delgado-Baquerizo et al., 2016; Louca et al., 2018). Understanding mechanisms by which microbial functions vary, including shifts in community composition, gene expression patterns, or density, will benefit studies in decomposition ecology and forensic science, in order to determine what changes are most meaningful during decomposition, and will aid in biomarker discovery for PMI estimation.

## Materials and Methods

### *S. aureus*/*C. perfringens* Preparation and Murine Inoculation

**FIGURE 1:**
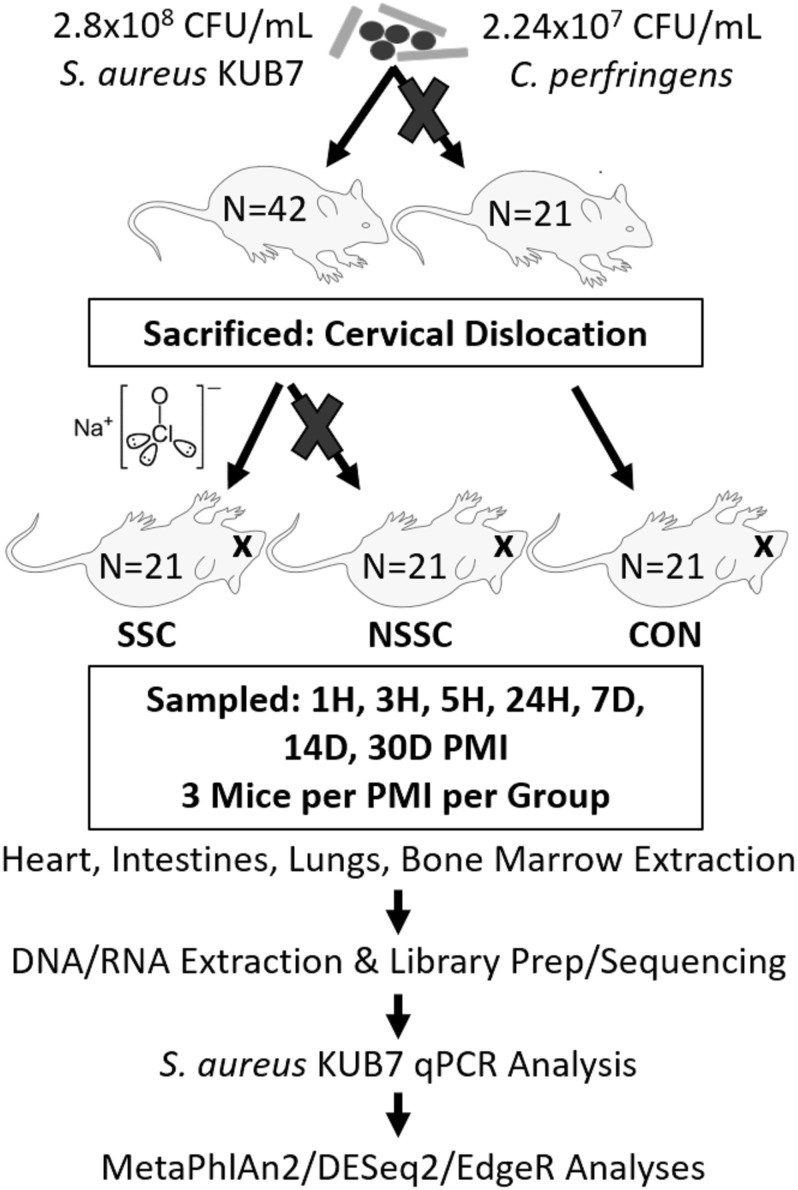
Experimental flow chart. A visual representation of the experimental design and downstream analyses. Mouse groups are represented as SSC = surface sterilized, colonized; NSSC = nonsurface sterilized, colonized; and CON = control.

A detailed description on the construction of *Staphylococcus aureus* KUB7 and *Clostridium perfringens* inoculums along with the murine inoculation, sacrifice, and organ harvest can be found in Burcham et al. (2016). An experimental procedure flow chart is detailed in Figure 1. Briefly, *S. aureus* KUB7 is a constructed strain that constitutively expresses a red fluorescent protein. *C. perfringens* type A strain WAL 14572 is non-fluorescent and was obtained from ATCC. *S. aureus* KUB7 and *C. perfringens* were grown to exponential phase in 100 mL tryptic soy broth (TSB) or reinforced clostridial medium (RCM), respectively. Sixty-four 1 mL cultures obtained from the original culture were pelleted and *S. aureus* KUB7 was resuspended in 7 μL TSB while *C. perfringens* was resuspended in 7 μL of RCM supplemented with 60 g/L sucrose. The resuspensions were combined and used for nasal inoculation through inhalation in 42 isoflurane sedated SKH-1 female mice (*N* = 42) obtained from Charles Rivers Laboratories with 21 mice not being inoculated as controls (*N* = 21) for a total of 90 mice (*N* = 63). The final inhalation inoculum was 2.8 × 10^8^ CFU/mL and 2.24 × 10^7^ CFU/mL for *S. aureus* KUB7 and *C. perfringens*, respectively. These two species have been shown to colonize living humans, albeit in separate niches, and have been shown to transmigrate and produce enzymes that break down tissues during decomposition (Kellerman et al., 1976; Melvin et al., 1984; Tuomisto et al., 2013; Burcham et al., 2016). We introduced chromosomally labeled red fluorescent *S. aureus* and non-labeled *C. perfringens* to the nasal pharynx and upper respiratory tract of mice by inhalation. This location was selected as it is a natural colonization site for *S. aureus* in humans and *C. perfringens* should not thrive in these oxygen rich environments. All animal experiments were conducted according to Mississippi State University IACUC approved protocol 14–102.

### Murine Sacrifice, Controlled Decomposition, and Organ Harvest

Twenty-four hours after inoculation, all mice were sacrificed by cervical dislocation, as previously described (Burcham et al., 2016). Twenty-one of the 42 inoculated mice were randomly chosen to be surface sterilized to disrupt the skin microbial communities. The surface sterilized mice were submerged up to below the mouth in a 10% bleach solution for 45 s avoiding sterilization of the mouth, nares, and ears to prevent the bleach solution from entering the body. The bleach solution was rinsed twice successively with distilled water. All 63 mice were individually placed in a Nalgene bottle top 0.2 μm filter container (Thermo Scientific) and sealed with Parafilm M to prevent environmental microbial and insect contamination. Mouse carcasses were allowed to naturally decompose within a bilaminar flow hood at ambient room temperature for up to 30 d during July 2015 in Starkville, MS, United States.

**FIGURE 2:**
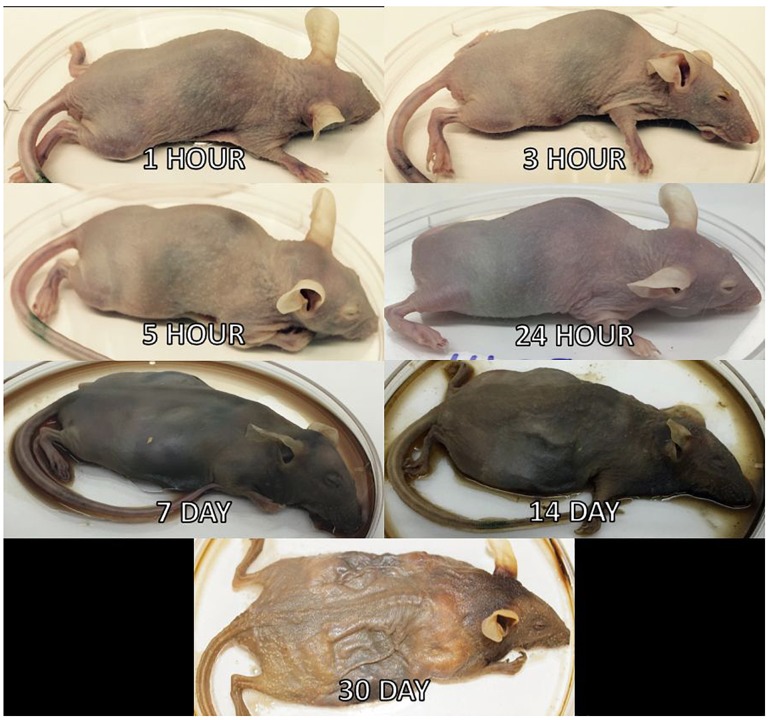
Mouse decomposition stages. Images of mice before dissection and organ harvest for their selected postmortem times to represent the decomposition stages present at each timepoint.

Three mice per treatment (control, inoculated with no surface sterilization, and inoculated with surface sterilization) were analyzed per time point (*T* = 1 h, 3 h, 5 h, 24 h, 7 d, 14 d, and 30 d postmortem). All tissue harvesting, and subsequent analyses were performed in a bilaminar flow hood under BSL2 and sterile conditions. A sterile scalpel blade was used to cut through the right hind leg and femur. The separated leg was used to obtain bone marrow from the femur using a sterile syringe containing nuclease-free water. A second sterile scalpel blade was used to dissect each mouse from the ventral side to remove the lungs, heart, and composite of the intestines using sterile forceps, individual for each organ. The bone marrow solution and each organ were transferred to individual 2.0 mL screw cap tubes. Each organ was crushed with a sterile cotton swab excluding the bone marrow solution. All organ swabs were swabbed on specialized agar plates for plate count determination discussed in Burcham et al. (2016). Afterward, RNAlater^®^ (Ambion) was added to each tube and the samples were stored in -20°C until nucleic acid extraction. Due to the variation of decomposition across individuals, some organs were no longer discernable in the later stages of decomposition and composite samples of the organ location were collected. Decomposition stages present at each postmortem timepoint are represented in Figure 2.

### Nucleic Acid Extraction and Purification

The following postmortem timepoints were chosen for sequencing to focus on the early decomposition processes: 1 h, 3 h, 5 h, 24 h, and 7 d. Therefore, to obtain both RNA and DNA from these samples, the dual extraction method using the TRIzol^TM^ Reagent (Thermo Fisher) standard issued protocol was performed on the preserved samples after being spun down and RNAlater^®^ removed. Briefly, 100 mg of tissue or the pelleted bone marrow was added to 1 mL of TRIzol^TM^ Reagent and a mix of 0.1/0.5 mm glass beads. The samples were homogenized in a bead beater with phase separation following chloroform addition. RNA was precipitated with isopropanol, washed with 75% ethanol, and resuspended in 50 μL of nuclease-free water, and incubated at 60°C for 15 min. The samples were purified using the PowerClean^®^ Pro RNA Clean-Up Kit (Qiagen), quantified fluorometrically using a Qubit 2.0^TM^ (Invitrogen), and then stored at -80°C. DNA was extracted during the TRIzol^TM^ Reagent protocol as described above. DNA within the sample interphase was precipitated with ethanol, washed with 0.1 M sodium citrate in 10% ethanol, washed with 75% ethanol, and resuspended in 0.6 mL of 8 mM NaOH. The samples were purified using a PowerClean^®^ Pro DNA Clean-Up Kit (Qiagen). All samples were quantified fluorometrically using a Qubit 2.0^TM^ (Invitrogen) and stored at -20°C. The non-sequenced timepoints (*T* = 14 d and 30 d) had DNA extracted using a modified protocol of that discussed in Williamson et al. (2014).

### *C. perfringens* WAL 14572 Detection

Difficulty in creating a fluorescently tagged *C. perfringens* strain resulted in detection of *C. perfringens* only in association with bacterial community analysis by metagenomic sequencing. Metagenomic sequencing analysis allowed determination of whether *C. perfringens* introduced nasally caused increased *C. perfringens* levels during early decomposition, particularly in less microbially rich organs, when compared to non-inoculated mice.

### *S. aureus* KUB7 Quantitative PCR Analysis

Transmigration of *S. aureus* KUB7 through body tissues as decomposition progressed was tracked at all postmortem times (1 h, 3 h, 5 h, 24 h, 7 d, 14 d, and 30 d) using qPCR by amplifying the red fluorescent protein gene incorporated in the genome of *S. aureus* KUB7. A 25 μL reaction was created using 3 μL template, 12.5 μL SsoAdvanced^TM^ Universal Probes Supermix (Bio-Rad), 1 μL forward primer (5′-TTGAAGGTGAAGGTGAAGGA-3′), 1 μL reverse primer (5′-TGCAAATGGTAATGGACCAC-3′), 2.5 μL FAM probe (5′-6FAMTGGAAGGTACACAAACAGCAAAAMGBNFQ-3′), and 5 μL nuclease-free water. The reaction was amplified using a Bio-Rad C1000 Touch^TM^ thermal cycler and measured with a Bio-Rad CFX96^TM^ real-time system with the following cycling conditions: 95°C for 10 min and (95°C for 15 s, 56°C for 30 s) × 40 cycles. Each sample was analyzed in duplicate to obtain an average CQ value and copies/run, which was extrapolated to obtain the mean genomic units of KUB7 per sample. The R statistical package was used to remove outliers in each organ as determined by Cook’s distance (R Core Team, 2013). The mean genomic units were log transformed, the mean log genomic units (logGU) ± standard error of the mean for each postmortem time for each organ were calculated. Wilcoxon rank sum significance testing between (non)surface sterilization did not show a difference between treatments (intestines: *p* = 0.79, bone marrow: *p* = 0.56, heart: *p* = 0.16, lungs: *p* = 0.84). Therefore, both treatments were combined and treated as the same for further testing. Significant differences between postmortem times were tested using a Kruskal–Wallis rank sum test for each organ. Significance was based on a *p* < 0.05.

### Metagenomic/Metatranscriptomic Library Preparation

The following postmortem timepoints were chosen for sequencing to focus on the early decomposition processes: 1 h, 3 h, 5 h, 24 h, and 7 d. Total DNA libraries were created using the NEBNext^®^ Ultra^TM^ DNA Library Prep Kit and NEBNext^®^ Multiplex Oligos (Dual Index Primers) for Illumina^®^ (New England BioLabs) protocols on all samples. Total RNA libraries were created using the NEBNext^®^ Ultra^TM^ RNA Library Prep Kit and NEBNext^®^ Multiplex Oligos (Dual Index Primers) for Illumina^®^ (New England BioLabs) protocols for use with purified mRNA or rRNA depleted RNA on the nonsurface sterilized and control samples excluding the lungs since they did not sequence well for metagenomic analysis. These protocols were chosen to maintain rRNA and mRNA within the RNA sample in order to preserve 16S rRNA genes and mRNA for both structural and differential transcript expression analysis. DNA samples were used for metagenomics community analysis.

### Whole Metagenome/Metatranscriptome Shotgun Sequencing and Processing

High-throughput whole metagenome and metatranscriptome sequencing was performed by St. Jude Children’s Research Hospital on an Illumina HiSeq2000 with 2 × 100 bp paired end (PE) read lengths. Sequences were initially trimmed by the sequencing facility using TrimGlare v0.4.2 (Krueger, 2015), but a more stringent quality trimming was performed using Trimmomatic v0.33 (Bolger et al., 2014). Metagenome sequences were trimmed to remove nucleotides in a four-position sliding window with an average phred33 score less than 28, and read lengths less than 36 bp. The trimmed metagenomic sequences were then used for bacterial community analysis. Metatranscriptome sequences were input in the SAMSA2 pipeline (Westreich et al., 2018). SAMSA2 was used to merge the paired-end sequences with PEAR v0.9.10 (Zhang et al., 2014), and then trimmed in a four-position sliding window with an average phred33 score less than 20, and read lengths less than 99 bp with Trimmomatic v0.3 (Bolger et al., 2014). SortMeRNA v2.1 (Kopylova et al., 2012) was used to remove bacteria/archaea 16S/23S rRNA genes and eukaryotic 18S/28S/5S/5.8S rRNA genes based on the documentation recommended SILVA and Rfam databases (Quast et al., 2013; Yilmaz et al., 2014; Kalvari et al., 2018). Sample identifiers and metadata can be found in Table 1.

**Table 1:** Sample metadata.

Sample	Treatment	Colonized	Sterilized	PMI	Organ	Number of DNA reads	Number of RNA reads	Genera richness
MG01	C	N	N	1H	BM	519	10642207	0
MG02	C	N	N	1H	HT	141	9424377	0
MG03	C	N	N	1H	INT	6869462	11198249	5
MG04	C	N	N	1H	LU	246592	NA	0
MG05	NS	Y	N	1H	BM	12541054	6174552	4
MG06	NS	Y	N	1H	HT	4069	4370991	0
MG07	NS	Y	N	1H	INT	13588235	9345718	9
MG08	NS	Y	N	1H	LU	7921779	NA	0
MG09	S	Y	Y	1H	BM	12371788	NA	2
MG10	S	Y	Y	1H	HT	114640	NA	0
MG11	S	Y	Y	1H	INT	23356918	NA	14
MG12	S	Y	Y	1H	LU	5764224	NA	0
MG13	C	N	N	3H	BM	3085317	6966014	0
MG14	C	N	N	3H	HT	68	6198445	0
MG15	C	N	N	3H	INT	1519870	10537046	3
MG16	C	N	N	3H	LU	552826	NA	0
MG17	NS	Y	N	3H	BM	9372874	6585375	2
MG18	NS	Y	N	3H	HT	11238	8625237	0
MG19	NS	Y	N	3H	INT	3748353	9724755	6
MG20	NS	Y	N	3H	LU	16267397	NA	0
MG21	S	Y	Y	3H	BM	12034029	NA	0
MG22	S	Y	Y	3H	HT	10885	NA	0
MG23	S	Y	Y	3H	INT	3403829	NA	3
MG24	S	Y	Y	3H	LU	13384061	NA	0
MG25	C	N	N	5H	BM	2082085	6939217	0
MG26	C	N	N	5H	HT	671145	7417681	0
MG27	C	N	N	5H	INT	12924794	9505797	6
MG28	C	N	N	5H	LU	667927	NA	0
MG29	NS	Y	N	5H	BM	14411164	6502696	3
MG30	NS	Y	N	5H	HT	1353231	6454480	1
MG31	NS	Y	N	5H	INT	4597616	5133461	7
MG32	NS	Y	N	5H	LU	12237082	NA	0
MG33	S	Y	Y	5H	BM	12515225	NA	0
MG34	S	Y	Y	5H	HT	14546830	NA	12
MG35	S	Y	Y	5H	INT	17560686	NA	10
MG36	S	Y	Y	5H	LU	20161875	NA	1
MG37	C	N	N	24H	BM	5704975	3622308	0
MG38	C	N	N	24H	HT	1583416	3309305	0
MG39	C	N	N	24H	INT	9105711	5814593	2
MG40	C	N	N	24H	LU	654955	NA	0
MG41	NS	Y	N	24H	BM	5137625	1608180	0
MG42	NS	Y	N	24H	HT	5126506	5397803	0
MG43	NS	Y	N	24H	INT	6336406	771048	3
MG44	NS	Y	N	24H	LU	11441973	NA	0
MG45	S	Y	Y	24H	BM	13481461	NA	0
MG46	S	Y	Y	24H	HT	4407690	NA	0
MG47	S	Y	Y	24H	INT	34742830	NA	4
MG48	S	Y	Y	24H	LU	20444728	NA	0
MG49	C	N	N	7D	BM	59584111	778479	2
MG50	C	N	N	7D	HT	7277358	3818602	3
MG51	C	N	N	7D	INT	142284	2124152	3
MG52	C	N	N	7D	LU	16679	NA	0
MG53	NS	Y	N	7D	BM	63145271	537412	2
MG54	NS	Y	N	7D	HT	10769919	3795345	1
MG55	NS	Y	N	7D	INT	6286983	5305095	10
MG56	NS	Y	N	7D	LU	11484731	NA	0
MG57	S	Y	Y	7D	BM	16535712	NA	1
MG58	S	Y	Y	7D	HT	9303940	NA	1
MG59	S	Y	Y	7D	INT	27283847	NA	21
MG60	S	Y	Y	7D	LU	13993375	NA	3


*Table demonstrates the sample identification with their treatment (C, control; NS, nonsurface sterilized; S, sterilized), if they were colonized (Y, yes; N, no), if they were sterilized (Y, yes; N, no), their PMI (1 h, 3 h, 5 h, 24 h, and 7 d), the organ the sample was obtained from (BM, bone marrow; HT, heart; INT, intestines; LU, lungs), the average number of DNA reads between paired-end reads, the number of RNA reads after paired-end merging, and the genera richness. Read counts are after quality control.*

### Metagenomic Bacterial Community Analysis

Relative abundance of the bacterial genera present in each metagenomic sample was determined using MetaPhlAn2 (Truong et al., 2015). MetaPhlAn2 uses roughly 1 million clade-specific markers from over 7500 species to characterize the microbial taxonomic profiles. Genera that constituted less than 3% of sample were grouped as rare taxa to reduce sampling noise, but were not grouped as rare taxa for community metrics so that rare taxa could be account for between test groups. The relative abundances were used to determine the log genera richness, Shannon diversity indices, and Bray–Curtis and binary Jaccard distance indices using the R statistical package vegan v2.5-1 (Oksanen et al., 2018). The metadata factors (colonization, sterilization, organ, and postmortem time) were used in a type-II Multivariate Analysis of Variance (MANOVA) additive model to test for differences in log genera richness, Shannon diversity indices, and Pielou’s evenness using the R statistical package car v3.0-0 (Fox and Weisbert, 2011). The Bray–Curtis and binary Jaccard indices were tested against the metadata factors in a type-II permutational MANOVA additive model using the R statistical package RVAideMemoire v0.9-69-3 (Herve, 2018). Both distance indices were calculated based on their nature to account for taxonomic abundances (Bray–Curtis) or to treat taxonomic data as presence–absence (Jaccard). Analysis of both distances is important as presence–absence data give more statistical weight to rare taxa while using abundances give more statistical weight to the taxa of higher abundance. Determination of the distance-based redundancy analysis (dbRDA) explanatory variables (metadata factors) was performed using forward selection with both distance indices after the recommended “method 1” transformation by Legendre and Anderson (1999). Forward selection is a method of stepwise regression which starts with an empty model and then adds the metadata factor which improves the model the most. This metadata factor addition continues with the remaining metadata until no more factors significantly improve the model. The forward selection determined significant explanatory variables were used to create dbRDA ordination plots with both distance indices and the significant explanatory variables as interactions. The genera driving the ordination distances were determined with permutation environmental fit by their *p*-value (*p* < 0.05) and *R*^2^. The dbRDA and environmental fit analyses were performed using the R statistical package vegan v2.5-1 (Oksanen et al., 2018).

### Transcript Annotation and Differential Expression Analysis

Ribosomal RNA depleted RNA transcripts were used in the SAMSA2 pipeline (Westreich et al., 2018). The reads were annotated using the DIAMOND sequence aligner to a database created from the March 2018 NCBI nr-protein database (Buchfink et al., 2015). The best protein hit for each read in the sample was aggregated for differential expression analysis using the R statistical packages edgeR v3.22.1 and DESeq2 v1.20.0 (Robinson et al., 2010; McCarthy et al., 2012; Love et al., 2014). Treatments were not differentiated, as community analysis showed no difference between colonization nor surface sterilization. In edgeR, the counts per million were computed and transcripts that were not present more than 1 CPM in at least two samples were removed to reduce noise. In DESeq2, transcripts that were not present in at least two samples with counts above 1 were removed. Normalization by library size was performed based on each packages’ recommended method. Normalization based on library size allows for samples with different numbers of RNA reads to be compared against each other without the read count differences affecting the results. This was especially important in our study as we found that our samples tended to decrease in RNA read abundance as decomposition progressed. EdgeR dispersion parameters for the negative binomial model estimations were determined individually per organ with bone marrow using bin-spline, heart using power, and intestines using spline. These dispersion methods were chosen independently for each organ based on which method provided a trendline which most fits the variation distribution. DESeq2 dispersions were estimated based on the default settings from the DESeq command. All models were created with no *y*-intercept and postmortem timepoint groups as the explanatory variable for each organ. EdgeR models were negative binomial generalized log-linear models (GLM) with quasi-likelihood and DESeq2 models were negative binomial GLMs with likelihood ratio tests between the full and reduced model without timepoints. For both methods, differential expression was tested to contrast the timepoint groups (early vs. middle, middle vs. late, early vs. late) for each organ and significantly differentially expressed transcripts were identified based on a Benjamini–Hochberg corrected *p*-value < 0.10 above 1 log2-fold-change threshold. Only transcripts determined to be significant by both methods were considered truly significantly expressed between the compared groups, to reduce procedural bias. Transcripts were annotated into pathways based on the KEGG and UniProt databases (Kanehisa and Goto, 2000; The UniProt Consortium, 2017).

## Results

### *S. aureus* KUB7 Quantitative PCR

**FIGURE 3:**
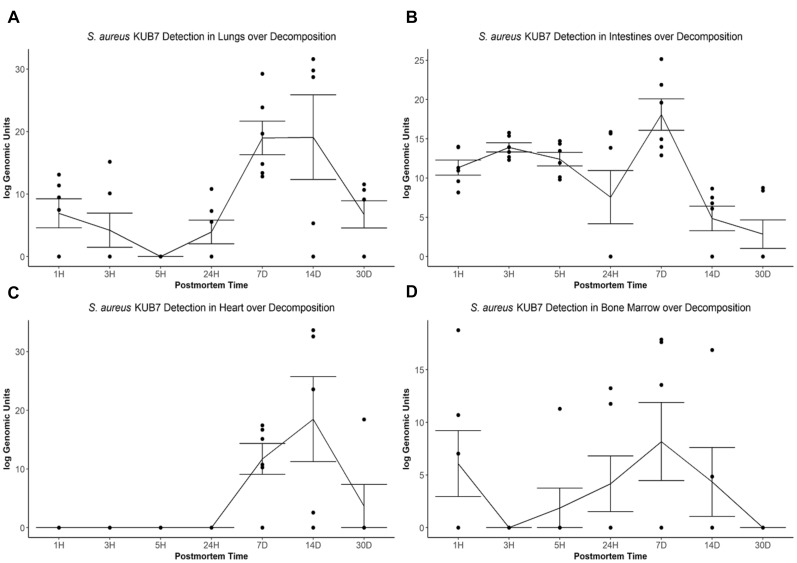
*S. aureus* KUB7 detection with qPCR in organs as decomposition progresses. The log genomic units are plotted on the *y*-axis and postmortem time on the *x*-axis. Each circle represents a sample and the line represents the mean (±SE) log genomic units of *S. aureus* KUB7 during decomposition in the **(A)** lungs, **(B)** intestines, **(C)** heart, and **(D)** bone marrow.

The *S. aureus* KUB7 mean log genomic units (logGU ± SE) in the lungs decreased from 6.91 ± 2.32 logGU to 4.21 ± 2.74 logGU then to 0 ± 0 logGU from 1 to 3 then 5 h after death, respectively. After 5 h, the mean logGU increased from 0 ± 0 logGU to 3.95 ± 1.90 logGU after 24 h, 18.97 ± 2.69 logGU after 7 d, and reached its maximum concentration (19.09 ± 6.77 logGU) after 14 d. Finally, the mean logGU decreased to levels similar to early timepoints of 6.75 ± 2.17 logGU at 30 d postmortem (Figure 3A). Overall, the lungs began with *S. aureus* KUB7 present then diminished within 5 h postmortem. Afterward, *S. aureus* KUB7 increased rapidly until returning to low levels after 14 d postmortem.

The *S. aureus* KUB7 mean logGU ± SE in the intestines stayed relatively consistent the first three timepoints (T = 1 h, 3 h, and 5 h) with logGU of 11.33 ± 0.95, 13.90 ± 0.58, and 12.40 ± 0.87, respectively. A decrease occurred at 24 h to 7.57 ± 3.40 logGU. The logGU increased to its maximum concentration after 7 d to 18.08 ± 2.0 logGU. *S. aureus* KUB7 detection decreased to below starting levels after 14 d (4.85 ± 1.57 logGU) to the minimum (2.86 ± 1.81 logGU) after 30 d postmortem time (Figure 3B). Overall, *S. aureus* KUB7 concentrations in the intestines during early postmortem times were relatively stable until exponential growth after 7 d, but then immediately began to decrease to levels below initial sampling.

*Staphylococcus aureus* KUB7 detection in the heart remained at zero until 7 d postmortem (11.72 ± 2.64 logGU) and increased to its maximum (18.49 ± 7.25 logGU) after 14 d. After 30 d, detection decreased to levels near zero (3.07 ± 3.07 logGU) (Figure 3C). Overall, the heart did not appear to be colonized, based on qPCR detection, by *S. aureus* KUB7 until 7 d postmortem, but once established, grew substantially until decreasing back to almost undetectable levels by 30 d postmortem.

*Staphylococcus aureus* KUB7 detection in the bone marrow started at 6.08 ± 3.13 logGU and decreased to 0 ± 0 logGU after 3 h. Detection increased to 1.88 ± 1.88 logGU after 5 h to 4.17 ± 2.64 logGU after 24 h and then to the maximum of 8.18 ± 3.71 logGU at 7 d. Detection decreased to 4.35 ± 3.27 logGU after 14 d then to 0 ± 0 logGU by 30 d postmortem (Figure 3D). Surprisingly, *S. aureus* KUB7 was detected almost immediately in the bone marrow after death, but then quickly decreased after 3 h until steadily increasing to its highest concentration after 7 d, then dissipating.

When testing for a significance difference between postmortem times in each organ using Kruskal–Wallis rank sum test based on logGU we were able to reject the null hypothesis that the mean logGU of all timepoints are equal in the lungs (χ^2^ = 19.71, *df* = 6, *p* = 0.003), intestines (χ^2^ = 23.94, *df* = 6, *p* = 0.0005), and heart (χ^2^ = 25.28, *df* = 6, *p* = 0.0003). We were not able to reject the null hypothesis for the bone marrow samples.

### Metagenomic Bacterial Genera Relative Abundance

**FIGURE 4:**
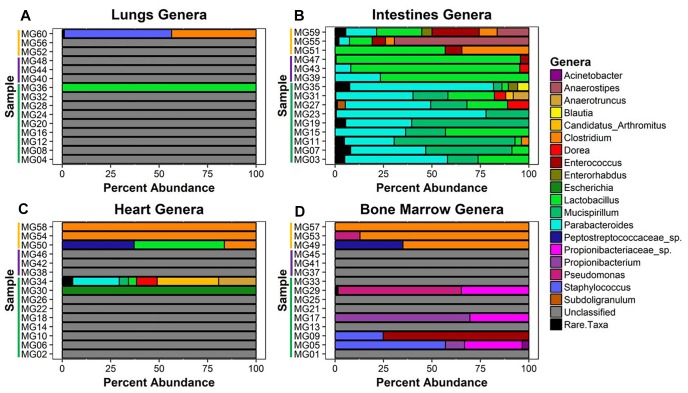
Bacterial genera relative abundance in samples at postmortem times. The bacteria genera detected in **(A)** lungs, **(B)** intestines, **(C)** heart, and **(D)** bone marrow samples are represented by their relative percent abundance on the *x*-axis with the sample on the *y*-axis. The samples are labeled by postmortem timepoint group with the color bars [green = early (1 h, 3 h, 5 h), purple = middle (24 h), and yellow = late (7 d)]. Genera that constituted less than 3% of sample were grouped as rare taxa to reduce sampling noise. Relative abundances were determined using MetaPhlAn v2.0.

Twenty-six unique genera were detected across the 60 samples for a total of 144 detections (min = 0, max = 21, mean = 2.4, *SD* = 4.08). Thirty-one samples did not match classified bacteria. These samples were pre-dominantly associated with early–middle (≤24 h) postmortem times in the lungs, bone marrow, and heart. In the lungs, only two samples provided community profiles (Figure 4A). Sample MG36 contained 100% *Lactobacillus* at 5 h and sample MG60 was made up of approximately 44% and 55% *Clostridium* and *Staphylococcus* at 7 d, respectively. As expected, the intestines provided the most robust abundance community profiles (Figure 4B). Early (≤5 h) postmortem times in the intestines showed dominating bacterial genera consisting of *Parabacteroides* (μ = 47.0%), *Mucispirillum* (μ = 29.6%), and *Lactobacillus* (μ = 14.4%). At 24 h, there was a decrease of relative abundance in *Parabacteroides* (μ = 10.5%), disappearance of *Mucispirillum* and increase of *Lactobacillus* (μ = 86.3%). At 7 d, *Lactobacillus* (μ = 30.6%) had decreased, allowing for the increase of *Anaerostipes* (μ = 28.6%), *Clostridium* (μ = 16.1%), and *Enterococcus* (μ = 13.3%). Bacterial genera within the heart were only detected at 5 h and 7 d (Figure 4C). At 5 h, sample MG30 showed 100% *Escherichia*, while MG34 showed a diverse community with the highest percentage of genera detected being *Candidatus Arthromitus* (31.7%), *Parabacteroides* (24%), *Anaerostipes* (19.3%), and *Dorea* (10.7%). At 7 d, the highest percentage of genera detected was *Clostridium* (μ = 72.1%), with *Lactobacillus* (μ = 15.5%) and Peptostreptococcaceae spp. (μ = 12.4%) being detected in one sample. In the bone marrow, four out of nine samples in early (≤24 h) postmortem times provided detected genera (Figure 4D). The early time group genera detected were Propionibacteriaceae spp. (μ = 10.6%), *Staphylococcus* (μ = 9.1%), *Propionibacterium* (μ = 8.8%), *Enterococcus* (μ = 8.3%), and *Pseudomonas* (μ = 7.1%). At 7 d, similar to heart sequences, Clostridium dominated the samples (μ = 84.0%), with Peptostreptococcaceae spp. (μ = 11.7%) and Pseudomonas (μ = 4.3%) also being detected.

### Metagenomic Bacterial Community Analyses

Type-II MANOVA additive model testing for log genera richness only showed a significant difference among organs (*SS* = 8.05, *df* = 2, *F* = 9.28, *p* = 0.002) with pairwise analysis determining differences between intestines–bone marrow (*p* = 0.01) and intestines–heart (*p* = 0.02). Type-II MANOVA additive model testing Shannon diversity indices only showed a significant difference among organs (*SS* = 1.71, *df* = 2, *F* = 4.07, *p* = 0.04) with pairwise analysis determining no significant pairs. Type-II MANOVA additive model testing squared Simpson’s diversity indices showed no difference. The type-II permutational MANOVA additive model for Bray–Curtis distance showed a difference between organs (*SS* = 1.74, mean sq. = 0.87, *df* = 2, *F* = 4.68, *p* = 0.001) and postmortem times (*SS* = 2.81, mean sq. = 0.70, *df* = 4, *F* = 3.79, *p* = 0.001). The type-II permutational MANOVA additive model for binary Jaccard distance showed a difference among organs (*SS* = 1.83, mean sq. = 0.92, *df* = 2, *F* = 4.19, *p* = 0.001) and postmortem times (*SS* = 2.2, mean sq. = 0.55, *df* = 4, *F* = 2.51, *p* = 0.001). It is important to note that bacterial diversity analyses comparing the treatments (control, inoculated with no surface sterilization, and inoculated with surface sterilization) showed no differentiation. We detected no difference between mice bacterial communities that were or were not surface sterilized nor did we detect a difference between the mice bacteria communities that were or were not inoculated with *S. aureus* and *C. perfringens*. Because of this we were able to combine the data across treatments to obtain higher sample sizes for comparing community structure and function across postmortem times and organs.

**FIGURE 5:**
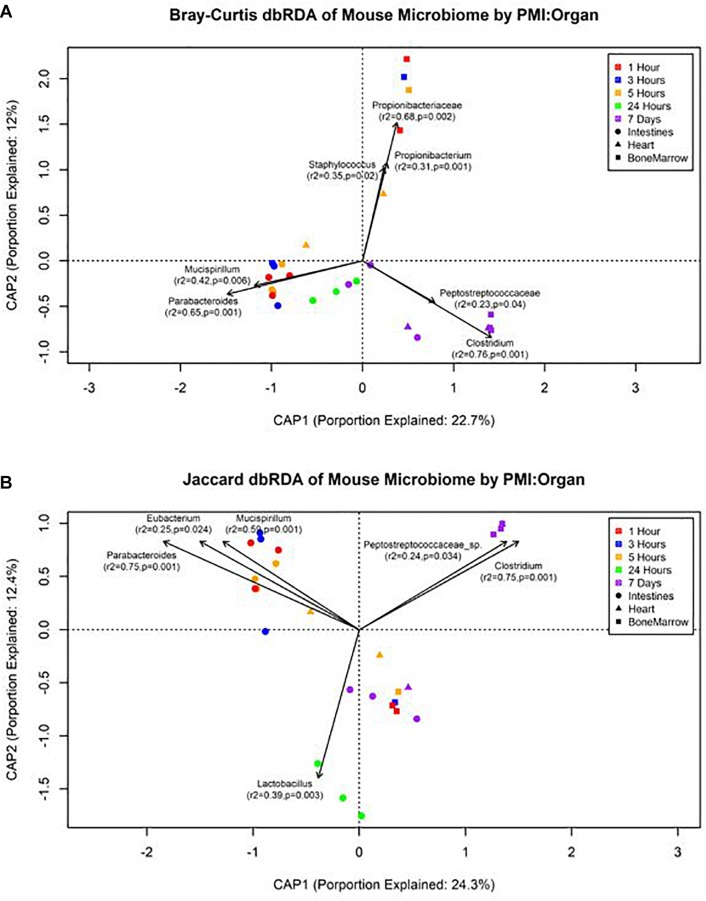
Distance-based RDA plots of the mouse organ microbiome over the postmortem interval. Distance-based RDA plots were created for both **(A)** Bray–Curtis and **(B)** Jaccard distances. Colors represent the postmortem intervals and shapes represent the organ from where the sample was obtained. Linear vectors were determined by genera with a significant environmental fit to the plot based on a *p* < 0.05.

The forward selection model for all metadata factors using dbRDA ordination on Bray–Curtis distances determined the explanatory variables to be PMI (*R*^2^ = 0.18, *df* = 4, AIC = 69.90, *F* = 2.45, *p* = 0.002) and organ (*R*^2^ = 0.32, *df* = 2, AIC = 66.32, *F* = 3.24, *p* = 0.002). These explanatory variables were treated as interactions to create the Bray–Curtis dbRDA ordination (Figure 5A). The environmental fit of the organ (*R*^2^ = 0.48, *p* = 0.001) and PMI (*R*^2^ = 0.46, *p* = 0.003) variables were significant with improved fits. Seven genera were identified to be structuring the ordination by environmental fit (Figure 5A). The forward selection model for all metadata factors using dbRDA ordination on Jaccard distances determined the explanatory variables to be PMI (*R*^2^ = 0.21, *df* = 4, AIC = 62.18, *F* = 2.76, *p* = 0.002) and organ (*R*^2^ = 0.35, *df* = 2, AIC = 58.43, *F* = 3.33, *p* = 0.002). These explanatory variables were treated as interactions to create the Jaccard dbRDA ordination (Figure 5B). The environmental fit of the organ (*R*^2^ = 0.32, *p* = 0.001) and PMI (*R*^2^ = 0.54, *p* = 0.001) variables was significant with the organ fit decreasing slightly and PMI fit improving. Six genera were determined to be driving the ordination by environmental fit (Figure 5B).

### Differential Expression

The edgeR mean transcript library sizes after filtering for the intestines was 24,055 (min = 10,549, max = 38,257, *SD* = 8702.23), the heart was 116,150 (min = 52,102, max = 191,191, *SD* = 47,094.28), and the bone marrow was 22,882 (min = 17,500, max = 84,116, *SD* = 25,909.62). The DESeq2 mean transcript library sizes after filtering for the intestines was 17,899 (min = 9570, max = 26,012, *SD* = 4920.05), the heart was 110,393 (min = 50,531, max = 185,198, *SD* = 46,014.13), and the bone marrow was 34,932 (min = 14,795, max = 79,693, *SD* = 24,868.38). Significantly differentially expressed transcripts for each method were determined, but only transcripts that were reported as significant by both edgeR and DESeq2 were considered significant to reduce program bias (Table 2). The intestines contained no significantly down-regulated or up-regulated transcripts.

**Table 2:** Number of transcripts detected from differential expression analysis.

Organ	Time comparison	Number of significantly up-regulated transcripts		Number of non-significantly expressed transcripts		Number of significantly down-regulated transcripts	
				
		DESeq2	EdgeR	DESeq2	EdgeR	DESeq2	EdgeR
Intestines	Early vs. middle	0(0)	0(0)	26,563	26,500	0(0)	2(0)
	Middle vs. late	0(0)	0(0)	26,563	26,501	0(0)	1(0)
	Early vs. late	0(0)	439(0)	26,563	26,055	0(0)	8(0)
Heart	Early vs. middle	86(0)	1(0)	27,968	24,623	52(0)	1(0)
	Middle vs. late	6024(3774)	6358(3774)	27,968	17,740	41(14)	527(14)
	Early vs. late	6023(3778)	8919(3778)	27,968	13,613	44(19)	2093(19)
Bone marrow	Early vs. middle	1(0)	0(0)	14,329	10,416	687(0)	0(0)
	Middle vs. late	36(3)	17(3)	10,278	14,326	483(44)	121(44)
	Early vs. late	16(7)	1022(7)	8632	14,689	704(268)	762(268)


*Time comparisons are separated by their postmortem time groups: early (1 h, 3 h, 5 h), middle (24 h), and late (7 d). The number of significant transcripts from each analysis method is shown with the number of common transcripts between the two methods being in parenthesis.*

**FIGURE 6:**
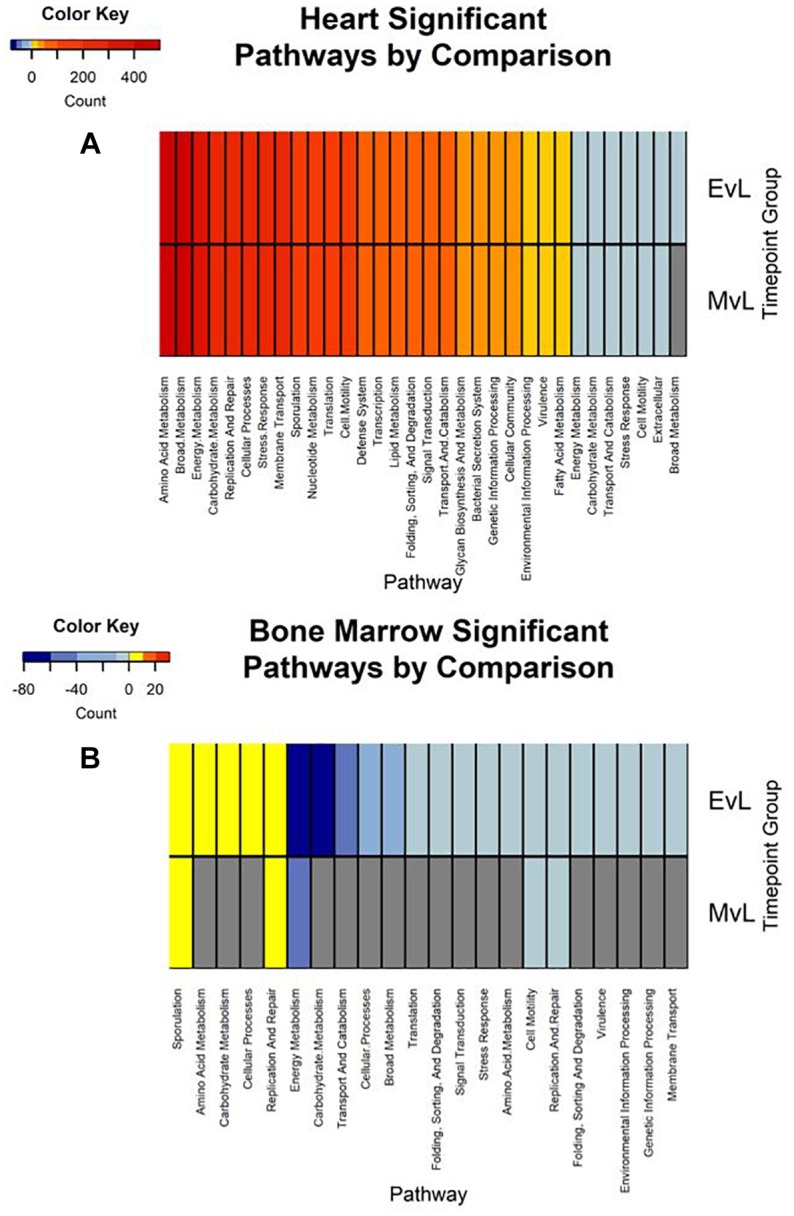
Heatmaps of the significant pathway regulation during timepoint group comparisons. Heatmaps for **(A)** heart and **(B)** bone marrow representing the transcript count of each pathway annotated from the significant transcripts of each comparison by color. Pathway is included on the *x*-axis and timepoint group (EvL = early vs. late, MvL = middle vs. late) on the *y*-axis. Down-regulated transcripts were considered negatives and up-regulated transcripts were considered positives. Pathways that were not detected in a comparison are in gray.

In total, the heart contained 58 significantly down-regulated transcripts, and 8305 significant up-regulated transcripts. Out of the significant transcripts in the heart, 25 of the 58 down-regulated transcripts and 753 of the 8305 up-regulated transcripts were annotated as hypothetical or ribosomal proteins. In total, the bone marrow contained 734 significantly down-regulated transcripts and 22 significant up-regulated transcripts. Out of the significant transcripts in the bone marrow, 422 of the 734 down-regulated transcripts and 12 of the 22 up-regulated transcripts were annotated as hypothetical or ribosomal proteins. A difference was detected in the heart and bone marrow between the early vs. late and middle vs. late group comparisons, while no difference occurred between the early and middle timepoint groups. In the heart, the pathway regulations were nearly identical with the majority of the pathways detected being up-regulated (*N* = 7552) (Figure 6A). Metabolic pathways were the most up-regulated with other notable pathways detected in abundance being stress response, sporulation, cell motility, and membrane transport. Few transcripts were significantly down-regulated (*N* = 33), but the most abundantly down-regulated were associated with metabolism (*N* = 15).

In the bone marrow, the majority of the significant transcripts occurred from a comparison of the early and late timepoint groups and most pathways were down-regulated (*N* = 312) (Figure 6B). Energy and carbohydrate metabolism were the most detected down-regulated pathways with other notable pathways being transport and catabolism, stress response, and cell motility. Few transcripts were significantly up-regulated (*N* = 10), though the most abundant pathway was sporulation (*N* = 3). A complete list of the individual significant transcripts with their logFC, FDR, NCBI nr protein annotations, and pathway annotations can be found in Online Supplementary Material (Supplementary Table S1).

## Discussion

The microbial changes that take place during decomposition have been previously studied with the intent to describe patterns that are consistent, reproducible, and precise for forensic evidence. Our data have shown that an individual *S. aureus* bacterial strain can be tracked as it migrates across organs in the body and behaves in a similar manner across different locations for when it reached maximum abundance and subsequently began to decline. *S. aureus* KUB7 and *C. perfringens* WAL 14572 introduction nor surface sterilization significantly altered the bacterial community structures. It is possible the introduction of *S. aureus* KUB7, while detectable through sensitive DNA techniques, was not at a high enough concentration to highly alter the existing community structure in a manner that would affect the diversity analyses. *C. perfringens*, while not detectable with qPCR, did not appear at levels higher than rare taxa during early decomposition in organs in which *C. perfringens* is not considered natural microbiota, such as the heart. A limitation to this approach of colonization is the difficulty to accurately assess the extent of competition that may have occurred between the natural microbiota and introduced species, and although the introduction of new species to any environment will inevitably affect the ecosystem, our results showed minimal microbial structure disruption when comparing the inoculated versus uninoculated mice suggesting that future transmigration studies could be performed without major concern for causing detrimental effects to the natural microbiota as long as researchers use appropriate organisms and colonization levels.

Additionally, the lack of significant microbial community alterations associated with internal organs by surface sterilization suggests that while external microbiota play a large role in skin decomposition, the breakdown of internal tissues relies predominately on internal microbes. For example, if after sterilization of the skin, internal organs have similar microbial profiles to non-sterilized hosts then the skin microbiome is not playing a large role in the internal organ decomposition. Alternatively, if skin-sterilized mice had drastically different microbial profiles internally than their non-sterilized counterparts, it would suggest that the microbial communities found in the internal organs during decomposition are heavily affected by the transmigration of skin microbes to the internal organs. Our results showed the former, suggesting that internal organs are decomposed primarily by internal microbes and not skin microbes. While this is not surprising, few studies to our knowledge have specifically tested this hypothesis. These results provide strength for the use of internal microbes as potential internal PMI biomarkers, and are useful for future studies that may aim to investigate transmigration and source tracking of specific internal microbes in relation to PMI.

Our transmigration tracking of inhaled *S. aureus* KUB7 showed similar overarching trends in the body as a whole, but with slight differences depending upon the organ (Figure 3). In the lungs (Figure 3A) and the heart (Figure 3C), *S. aureus* KUB7 reached its highest count at 14 d, while in the intestines (Figure 3B) and the bone marrow (Figure 3D) the highest count was reached at 7 d. We expected the lungs and the intestines to contain *S. aureus* KUB7 immediately after death as these sites were most likely to be colonized following inhalation, and sometimes leading to intake of *S. aureus* KUB7 down the esophagus. The decrease of *S. aureus* KUB7 in the lungs immediately following death could be attributed to competition with other microbial species, an increase in host immune gene transcripts which occurs up to 24 h postmortem, and/or lack of oxygen as blood flow and breathing ceased, though further research is needed to discover the specific mechanisms taking place in our system (Coleman et al., 1983; Pozhitkov et al., 2017). *S. aureus* is a facultative anaerobe that has been shown to have much slower growth in the absence of oxygen, though the lack of oxygen does not completely stop growth (Coleman et al., 1983; Belay and Rasooly, 2002). This may account for the decrease immediately following death, as metabolism shifted and a lag of growth occurs. The spike of growth from 7 to 14 d is likely due to the reintroduction of oxygen, being accustomed to an anaerobic environment, complete immune system loss, or increase of nutrients present after tissue breakdown (Janaway et al., 2009; Hyde et al., 2013; Crippen et al., 2015; Pozhitkov et al., 2017). The same reasoning can be used to explain the trend found in the intestines as the growth spike occurs at the same time, but the absence of a dip immediately following death may be attributed to the fact that the environment in the intestines is already anaerobic and a drastic shift in oxygen availability did not occur coupled with the fact the *S. aureus* KUB7 present had likely already adjusted to this environment during living colonization. Although it is important to note that we did not generate oxygen level data from our model and could not confirm the aerobic and anaerobic shifts. Furthermore, the presence of non-circulating, active but decreasing host apoptotic cells and neutrophils along with increased immunity gene transcripts between early and late timepoints could also potentially account for fluctuations in *S. aureus* KUB7 concentrations (Heimesaat et al., 2012; Pozhitkov et al., 2017). All organs returned to bacterial levels either similar to or below 1 h postmortem levels by the end of 7 d.

The bone marrow and heart trends are interesting as they do not represent locations where colonization should have occurred from initial host inhalation, and in fact should represent sterile locations during life that are colonized through transmigration after death. Interestingly, *S. aureus* KUB7 was detected within the first hour in the bone marrow, possibly resulting from rapid transmigration or an artifact from the skin when the femur was cut. If rapid transmigration occurred, then *S. aureus* KUB7 was not able to thrive in the early bone marrow environment leading to the immediate decrease at 3 h. However, transmigration began slowly around 5 h until reaching its highest concentration after 7 d. The heart had no detection of *S. aureus* KUB7 until 7 d postmortem and reached maximum detected abundance at 14 d. Suggesting *S. aureus* KUB7 migrated to the heart sometime after 24 h, though it is not clear exactly when it occurred. The 14 d peak may be due to the lag in time between transmigration and heart colonization, as the organ is presumed sterile during host life. The decline seen at the later timepoints is likely due to nutrient limitations or toxicity. However, determining the window where migration into the heart can be pinpointed may serve as a useful biological marker for narrowed PMI estimation ranges.

Bacterial community and differential expression analyses aimed to (1) strengthen existing knowledge of community shifts in vertebrate intestines, (2) provide data toward community shifts in underexplored organs, and (3) identify cellular processes and metabolic changes taking place from early to late decomposition. We provide one of the first sets of metatranscriptomic analyses of these communities showing genes active across decomposition time. Functional analyses performed by other groups have been based on 16S rRNA predictive function, but it is important to study active gene function because, while it may be predicted a gene is expressed, it must be confirmed by expression data if functional biomarkers can be discovered. Additionally, the ability to determine functional activity in a microbial community has the opportunity to by-pass one of the main concerns of solely monitoring the postmortem microbial structure. This concern focuses on the fact that human microbial profiles are unique to the individual and while one individual may carry a certain species of bacteria, another person may not. This can be concerning if using detailed species markers that may not be found ubiquitously across human populations. Monitoring the function of microbes during decomposition may circumvent this concern because, while the individual species may change between people, the functions needed to survive during each PMI should be redundant. For example, the functional pathways needed to utilize decomposition nutrients and survive oxygen fluxes will be highly expressed no matter the individual organism. Another consideration is that species in microbial communities may have dissimilar functional roles during decomposition, that might be obscured while analyzing functional potential from metagenomic data. Therefore, integrating community composition with active gene function may provide evidence to the state of the microbial decomposition ecology across PMIs that are more concrete and less biased to the individual. This coupling of structure and function data, as we have shown, may also yield a predictive framework for determining associations between community structure and function during decomposition, with utility for forensic science. Therefore, we suggest that more empirical data should be gathered linking microbial functional groups and genes with community structure in studies of decomposition ecology.

Our data suggest genera richness and Shannon diversity indices vary by organ. Bray–Curtis and Jaccard indices were affected by both organs and postmortem time. This is not surprising as the intestines are a natural location of high levels of microbial diversity compared to other organs, and as decomposition occurs these less diverse organs begin to increase in diversity as transmigrations occur due to the increase of nutrients and lack of immune system after 24 h (Pechal et al., 2014; Guo et al., 2016). These microbial shift trends have been shown in both animal and human models (Heimesaat et al., 2012; Hyde et al., 2013). We found that Bray–Curtis dissimilarity index ordination fit the community profile and genera environmental fit better than Jaccard (Figure 5). Jaccard indices treat data as presence–absence, while Bray–Curtis indices accounts for genera richness, which is needed when comparing organs and postmortem times that have been previously shown to vary drastically in their diversity and richness.

We detected distinct microbial changes in the community structure, which corroborate shifts seen in other studies (Metcalf et al., 2013; Tuomisto et al., 2013; Pechal et al., 2014; Hauther et al., 2015). In particular, the ratio decreased of *Parabacteroides* and increase of *Lactobacillus* in the first 24 h followed by the increased ratio of *Enterococcus* and *Clostridium* in late stages is a common postmortem trend (Figure 4B). The Bray–Curtis ordination (Figure 5A) similarly shows the transition of the intestinal community from *Parabacteroides* to *Clostridium*. However, we did not detect any significantly differential expressed transcripts within the timepoint group comparisons for the intestine microbial communities (Table 2). The cellular pathways needed to survive in the intestines may be relatively consistent during the first 7 d, but then community turnover in the intestines may be attributed to factors involved in abilities to use alternate sources to maintain existing pathways, outcompete for nutrients, or replicate faster.

Due to poor sequence coverage or bacterial abundance lower than the needed threshold for quality sequencing, only two lung samples (MG36 and MG60) and two heart samples (MG30 and MG34) from the first 24 h could be classified for structure profiles (Figure 4A,C). The two 5 h cardiac samples were drastically different because MG30 only has a single genus detected (*Escherichia*), while MG34 and contained six genera that were not considered rare taxa. The 7 d heart samples were mostly predominated by *Clostridium* with one sample containing *Lactobacillus* and Peptostreptococcaceae; all of which are common Gram-positive gut bacteria associated with decomposition (Pechal et al., 2014; Javan et al., 2017). The heart contained the most differentially expressed transcripts (Table 2). Over 99% of the transcripts were up-regulated from ≤24 h to 7 d postmortem. This activity is likely due to the influx of microorganisms from transmigration leading to an increased usage of motility and metabolism pathways to use nutrients in the new environment (Figure 6A) as some organisms, such as some species of *Clostridium*, are motile and replicate extremely fast. These organisms could also have migrated to the heart between 24 h and 7 d, leading to nutrient depletion after 7 d with an increased stress response and sporulation to prepare for dormancy.

The bone marrow had bacterial classifications in five samples ≤24 h postmortem (Figure 4D). The majority of these early-detected genera are associated with the skin microbiome. Since early timepoints contained multiple genera associated with the skin microbiome, it is likely early bone marrow samples obtained these genera during dissection, but it cannot be fully ruled out that early transmigration took place. As with the heart, the bone marrow 7 d samples were dominated by *Clostridium*. However, opposed to the heart, the bone marrow’s differentially expressed transcripts were predominately down-regulated (312 of 322 classified genes) (Table 2). These down-regulated transcripts were primary attributed to energy metabolism, carbohydrate metabolism, and transport and catabolism (Figure 6B). The early bone marrow genera can survive in oxygenated environments (i.e., *Propionibacterium*) so that anaerobic shifts would cause oxidative energy metabolism pathways to be down-regulated as they switch to the preferred anaerobic energy metabolism pathways. Additionally, these profiles could result from late-arriving genera (i.e., *Clostridium*) having to use carbohydrates not available in the early stages of decomposition, and obtaining energy anaerobically as the oxygen availability shifted from aerobic to anaerobic.

We also detected up-regulation of sporulation in marrow samples during late decomposition, suggesting a need to prepare for dormancy, similar to the heart community. The Bray–Curtis ordination (Figure 5A) separated the early bone marrow samples from the rest of the organs due to the early classification of potential skin microbes, but showed similarities in bone marrow and heart microbial communities. A homogenization of microbial communities (*Clostridium* begins to dominate) probably occurs as organ communities become more similar due to the decomposition process. However, a similar trend was not clearly visualized by the Jaccard ordination (Figure 5B), as early bone marrow and late intestines were similar in Jaccard distance though not reflected in the detected microbial communities from each organ at those times. These conflicting data reflect an important point of consideration when dealing with different organ ecosystems using a presence–absence model, as opposed to taking in account the genera richness. This suggests comparing ecosystems suspected to vary in microbial abundance and diversity should be compared using methods that take into account richness, to not overinflate rare taxa (Lozupone et al., 2007).

Additionally, it is important to note that the decomposition process is highly affected by environmental factors and host individuality. In this study, many of these factors were controlled by using a genetically identical model, eliminating scavengers, and maintaining ambient temperature/moisture. But in a more natural setting these factors alter decomposition rates making PMI estimation more difficult. Although the time it takes a cadaver to progress through the stepwise patterns of decomposition can vary, the process itself is rather ubiquitous, with the exception of extreme cases. Because of this, it is suspected that metabolic activities of microorganisms associated with decomposition may be similar across ecosystems and individuals during decomposition with little variance. For example, the need to transcribe genes for survival during oxygen fluxes is universal no matter the specific species present. Our data show that the functional profiles of these microbial communities are PMI dependent and coupled with community structural data may provide better insight on the mechanisms of microbially mediated decomposition, though the impact of environmental factors on these functional changes are still poorly understood.

We have also shown community structure results in our system that correlate with results that have been shown in work by other researchers using animal and human models in multiple geographic locations (Pechal et al., 2014; Guo et al., 2016; Metcalf et al., 2016; Javan et al., 2017). This corroboration by multiple, independent sources is a critical step for future implementation of microbiological assessment to the PMI in forensic cases (Burcham et al., 2016). However, abiotic factors not measured in this study, such as pH and oxygen levels, should be assessed in further studies to expand on the microbial physiological responses of these communities. Lastly, we have performed one of the first studies combining bacterial taxonomic and metatranscriptomic analyses as decomposition occurs, which includes detailed exploration of both commonly and underreported microbial communities of the postmortem microbiome. From these, we have shown distinct changes in microbial community structure and function during decomposition succession. Microbial community structure in conjunction with community functioning are imperative to understand as we explore in-depth analysis of transmigration and microbial succession of commensal organisms in response to environmental disturbances within the once living host. We have provided a broad overview of the metabolic and stress response changes taking place during decomposition, along with the individual transcript fold changes, so that future research can narrow its scope to clusters of genes, along with associated community composition, with potential for biomarkers to aid in PMI determination.

## Ethics Statement

This study was carried out in accordance with the principles of the Basel Declaration and recommendations of the Mississippi State University Institutional Animal Care and Use Committee approved (protocol 14–102), Institutional Biosafety Committee (protocol 009–14), and approved by both the University Institutional Animal Care and Use Committee and Institutional Biosafety Committees.

## Author Contributions

ZB performed quantitative and computational analysis. JP, CS, MB, and HJ conceived of the presented idea, obtained funding for the project, and supervised the findings of this work. JB created the fluorescent strain utilized in this project. JR was responsible for obtaining the metagenomic and metatranscriptomic sequencing data. All authors discussed the results, reviewed, and contributed to the final manuscript.

## Conflict of Interest Statement

The authors declare that the research was conducted in the absence of any commercial or financial relationships that could be construed as a potential conflict of interest.
